# Nursing students’ knowledge and attitude in relation to COVID-19 prevention behavior

**DOI:** 10.1590/0034-7167-2022-0588

**Published:** 2023-08-07

**Authors:** Vhania Dhominica Bani, Pinka Kinanthi Gusti, Marlita Enjel Rawul, Martina Pakpahan, Ester Silitonga

**Affiliations:** I Universitas Pelita Harapan. Tangerang, Indonesia

**Keywords:** COVID-19, Health Behavior, Knowledge, Attitude, Nursing Student., COVID-19, Comportamento Relacionados com a Saúde, Conhecimento, Atitude, Estudantes de Enfermagem., COVID-19, Conductas Relacionadas con la Salud, Conocimiento, Actitud, Estudiantes de Enfermería.

## Abstract

**Objectives:**

to determine the correlation between nursing students’ level of knowledge and attitudes toward COVID-19 prevention behavior.

**Methods:**

a cross-sectional study was carried out at the Private University in Indonesia. Accidental sampling was used to select 188 third-year bachelor’s nursing students as respondents. Data collection was conducted through an online questionnaire (Cronbach’s Alpha 0.799-0.959) consisting of 27 questions. The data were analyzed descriptively and inferentially.

**Results:**

as many as 49.5 % of respondents had high knowledge, 98.4 % had a positive attitude, and 89.9 % had positive behavior in preventing COVID-19 transmission. The Chi-square test revealed that knowledge has no correlation with COVID-19 prevention behavior (p-value 0.864), but attitude did (p-value 0.027).

**Conclusions:**

nursing students’ attitudes are related to behavior in preventing COVID-19. Nursing students are expected to maintain positive attitudes and behaviors toward COVID-19 prevention as future health workers at the forefront of health services.

## RESUMO

**Objetivos:**

determinar a correlação entre o nível de conhecimento e as atitudes dos estudantes de enfermagem em relação ao comportamento de prevenção à COVID-19.

**Métodos:**

estudo transversal realizado em Universidade Privada da Indonésia. Amostragem acidental foi usada para selecionar 188 estudantes do terceiro ano do bacharelado em enfermagem como entrevistados. A coleta de dados foi realizada por meio de questionário online composto por 27 questões (Alfa de Cronbach 0,799-0,959). Os dados foram analisados de forma descritiva e inferencial.

**Resultados:**

do total de entrevistados, 49,5% possuíam alto conhecimento, 98,4% tinham uma atitude positiva, e 89,9% tinham comportamento positivo na prevenção da transmissão à COVID-19. O teste do Qui-quadrado revelou que o conhecimento não tem correlação com o comportamento de prevenção à COVID-19 (valor p 0,864), mas a atitude tem (valor p 0,027).

**Conclusões:**

as atitudes dos estudantes de enfermagem estão relacionadas com o comportamento na prevenção da COVID-19. Espera-se que os estudantes de enfermagem mantenham atitudes e comportamentos positivos em relação à prevenção da COVID-19 como futuros profissionais de saúde na linha de frente dos serviços de saúde.

## RESUMEN

**Objetivos:**

determinar la correlación entre el nivel de conocimiento y las actitudes de los estudiantes de enfermería en relación a la conducta de prevención de la COVID-19.

**Métodos:**

estudio transversal realizado en una Universidad Privada de Indonesia. Se utilizó un muestreo accidental para seleccionar a 188 estudiantes de tercer año de enfermería como encuestados. La recolección de datos se realizó mediante un cuestionario en línea que consta de 27 preguntas (alfa de Cronbach 0.799-0.959). Los datos fueron analizados de forma descriptiva e inferencial.

**Resultados:**

el 49,5 % de los encuestados tenía conocimientos altos, el 98,4 % tenía una actitud positiva y el 89,9 % tenía un comportamiento positivo en la prevención de la transmisión de la COVID-19. La prueba de Chi-cuadrado reveló que el conocimiento no tiene correlación con el comportamiento de prevención de COVID-19 (valor de p 0,864), pero la actitud sí (valor de p 0,027).

**Conclusiones:**

las actitudes de los estudiantes de enfermería están relacionadas con el comportamiento en la prevención del COVID-19. Se espera que los estudiantes de enfermería mantengan actitudes y comportamientos positivos hacia la prevención de COVID-19 como futuros profesionales de la salud en la primera línea de los servicios de salud.

## INTRODUCTION

COVID-19 is caused by SARS-CoV-2, which causes mild to severe acute respiratory system disorders such as fever, coughing up phlegm, headache, shortness of breath or breathing difficulties, fatigue, and new loss of taste or smell^(1)^. COVID-19 was declared a global pandemic by the World Health Organization (WHO) on March 11, 2020. According to the WHO (2021), until October 5, 2021, there were 235,175,106 confirmed cases of COVID-19 in the world and 4,806,841 deaths, while in Southeast Asia there were 43,189,962 confirmed cases of COVID-19 and Indonesia ranks first in Southeast Asia with 4,221,610 cases and 142,338 deaths^(2)^.

As of October 6, 2021, Banten province had the seventh-highest number of positive COVID-19 cases among Indonesia’s 33 provinces, with 131,727 positive cases and 2,676 deaths. Furthermore, it is known that there are 26,866 positive COVID-19 cases in Tangerang Regency, with a mortality prevalence of 392 people, of which 5,072 people were confirmed positive for COVID-19 and 66 deaths occurred in Kelapa Dua District^(3)^. In response to the high number of COVID-19 cases, the Indonesian government established a national health protocol that includes washing hands with soap, wearing masks, and practicing social distancing to avoid crowds, all of which are aimed at breaking the COVID-19 transmission chain^(4)^.

According to official data obtained from a private university in Tangerang City, 62 of 330 third-year nursing students were confirmed positive for COVID-19 between January and September 2021. These students were actively practicing, whether it was in hospitals, on practical assignments, or assisting with COVID-19 vaccinations, which necessitated going in and out of dorms. According to preliminary research, third-year nursing students had high knowledge and positive attitudes, but their behaviors did not reflect efforts to break the chain of transmission of the COVID-19 virus. They do not always wear masks in a public areas or outside, do not always practice hand hygiene, do not always practice social distancing, and do not always change clothes or take a shower after traveling. This increases the possibility of new COVID-19-positive cases among nursing students at a private university in the future. Furthermore, if the student continues with the negative behavior, it may be harmful to others.

According to the Integrated Behavior Model, several factors influence a person’s behavior, including demography, knowledge, attitudes, habits, norms, media, and environments^(5)^. Behavioral changes resulting from a person’s self-awareness are critical to health maintenance and will last longer^(6)^. Health students are expected to be proactive in preventing COVID-19 transmission, even more than they are expected to be role models for the community because health workers are on the front lines of COVID-19 services and act as agents of change to break the chain of virus transmission within the scope of healthcare facilities.

Yanti *et al*. discovered that Indonesian communities have good knowledge, attitude, and behavior toward social distancing to prevent virus transmission in 29 provinces in Indonesia^(7)^. Respondents with good knowledge demonstrated positive attitudes and behavior, while respondents with positive attitudes demonstrated good behavior^(7)^. A study by Puspitasari *et al*. in April 2020 revealed that healthcare workers, medical students, and populations in the United States, the United Kingdom, Italy, Jordan, and China have positive knowledge, optimistic attitudes, and good practices^(8)^. Knowledge had a direct impact on both attitudes (such as perceived risk and efficacy belief) and practices (e.g., personal hygiene and social distancing)^(9)^. Knowledge, year of study, perceived health status, and country of birth, all have an impact on practices in nursing students^(10)^.

Nursing students are demonstrated to maintain positive attitudes and behaviors toward COVID-19 prevention as future health workers at the forefront of health services. As a result, the authors want to investigate nursing students’ COVID-19 prevention knowledge, attitudes, and behavior. Understanding nursing students’ COVID-19 prevention knowledge, attitudes, and behavior can aid in predicting the outcome of planned behavior. If their attitudes and behavior can be identified, educational institutions and practice sites can use this information to provide relevant training, health resource, adequate facilities, policies, and monitoring during the pandemic.

## OBJECTIVES

To determine the correlation between nursing students’ level of knowledge and attitudes toward COVID-19 prevention behavior.

## METHODS

### Ethical aspects

The study has been ethically approved by the ethics committee of the Faculty of Nursing, Universitas Pelita Harapan, whose approval letter is attached to this submission. The study was conducted with ethical principles such as informed consent, confidentiality, and justice. The research subjects are completely free in determining whether to be a respondent or not.

### Study design, location, and time period

The study employed a cross-sectional design, in which all variables were measured concurrently. The study was carried out at a private University in Tangerang, between February and April of 2022.

### Population and sample

This study’s population consisted of 330 third-year bachelor’s nursing students from a private university in Tangerang. The third-year bachelor’s nursing students were divided into seven groups, with groups one serving as the respondent to the validity and reliability test and groups two through seven serving as the study respondent. The Slovin formula yielded a sample size of 171 respondents, with an additional 10% added to anticipated respondents who filled out incomplete data, for a total of 188 respondents. The accidental sampling method was used to collect 188 samples for this study. The respondent was third-year bachelor’s nursing students and was not involved in the validity and reliability test. Reducing the possibility of selection bias in accidental sampling, the researcher includes key questions based on the respondent’s criteria in the questionnaire as validation when selecting samples.

### Study protocol

The independent variables in this study were knowledge and attitude, and the dependent variable was behaviors. The instrument was an online questionnaire created with Google Forms. The authors modified a questionnaire from Wairata’s questionnaire (2020) to assess this study’s respondents’ knowledge, attitudes, and behaviors^(11)^. Following approval from the ethics committee, the researcher conducted a validity and reliability test for the questionnaire. Only those questions in Waitrata’s questionnaire that passed the VR test were used in this study. An online questionnaire passed the validity and reliability tests to 30 respondents, with R count 0.381 - 0.703 and Cronbach’s Alpha 0.799 for the knowledge variable; R count 0.639 - 0.907 and Cronbach’s Alpha 0.959 for the attitude variable; R count 0.328 - 0.643 and Cronbach’s Alpha 0.8 for the behavior variable. The questionnaire has 27 questions, with eight for the knowledge variable (multiple choices), nine for the attitude variable (Likert scale: strongly disagree, disagree, agree, and strongly agree), and ten for the behavior variable (Likert scale: never, rarely, often, always).

### Analysis of results and statistics

This study used descriptive (frequency & percentage) and inferential analysis using the Chi-square test. The data examined is categorical. Knowledge is divided into three categories: low, moderate, and high; attitude is divided into two categories: negative and positive; and behavior is divided into two categories: negative and positive. Incomplete data will be excluded and will not be analyzed further. The authors use the SPSS version 28 software as a testing tool for the analysis.

## RESULTS

Data was collected from 188 respondents, and all data were complete or not missing. Tables 1-4 present the study’s findings. The univariate analysis included respondent characteristics, knowledge, attitudes, and behaviors to prevent COVID-19 transmission. The bivariate analysis examined the relationship between knowledge and behavior in preventing COVID-19 transmission and the relationship between attitudes and behavior in preventing COVID-19 transmission.

**Table 1:** Distribution of Respondents’ Characteristics (N=188)

Characteristics	n	%
Gender Man Woman	47 141	25 75
Age < 20 years old ≥20 years old	12 176	6.38 93.62

Table 1 shows that most respondents were aged ≥ 20 years old (93.62%) and women (75%). It is known that the majority of respondents, as many as 185 (98.4%) have a positive attitude, 169 (89.9%) have a positive behavior and 93 (49.5%) have high knowledge (Table 2).

**Table 2:** Description of Respondent’s Knowledge, Attitude, and Behavior in The Prevention of COVID-19 (N=188)

Variable	n	%
Knowledge Low Moderate High	14 81 93	7.4 43.1 49.5
Attitude Negative Positive	3 185	1.6 98.4
Behavior Negative Positive	19 169	10.1 89.9

According to Table 3, 84 (44.7%) respondents have a high level of knowledge and positive behavior in preventing COVID-19 transmission. Meanwhile, only two (1.1%) respondents have low knowledge and negative behavior in preventing COVID-19 transmission. Furthermore, p-value of 0.864 was obtained, indicating no correlation between knowledge and behavior to prevent COVID-19 transmission.

**Table 3:** The Correlation Between Knowledge and Behavior in The Prevention of COVID-19 (N=188)

Knowledge	Behavior						
	Positive		Negative		*p* value	OR	95*%*CI
	n	%	n	%			
Low	12	6.4	2	1.1			
Moderate	73	38.8	8	4.2	0.864	0.64	0.12-3.39
High	84	44.7	9	4.8		0.98	0.36-2.67

Table 4 shows that 168 (89.4%) respondents have a positive attitude and are acting positively to prevent the transmission of COVID-19. Meanwhile, only two (1.1%) respondents have negative attitudes and behaviors toward preventing COVID-19 transmission. Furthermore, the p-value was 0.027, indicating a correlation between respondents’ attitudes toward preventing COVID-19 transmission behavior. Table 4 also shows that the Odds Ratio (OR) was 19.76 (95% CI: 1.70-229.44). This means that nursing students with a positive attitude toward the prevention of COVID-19 are 19.76 times more likely to behave positively in the prevention of COVID-19 than nursing students with a negative attitude.

**Table 4:** The Correlation Between Attitudes and Behavior in The Prevention of COVID-19 (N=188)

Attitude	Behavior						
	Positive		Negative		*p* value	OR	95*%*CI
	n	%	n	%			
Negative	1	0.5	2	1.1	0.027	19.76	1.70-229.44
Positive	168	89.4	17	9			

## DISCUSSION

According to the study’s findings, most respondents were women, aged 20 and up. Some studies discovered that gender, age, length of study, institution type, and institutional status all significantly impacted the practice domain^(12-13)^. Female students are more considerate than male students, students over the age of twenty possess better habits than students under that age, and students at public institutions have significantly improved practices than students at private institutions^(12-13)^. Women are more likely to regard COVID-19 as a serious health issue, support restraint measures, and comply with public health and social distancing measures^(14)^.

Education, experience, and the availability of information sources all have an impact on one’s level of knowledge^(15)^. The study discovered that half of the third-year nursing students had high knowledge of COVID-19 prevention behavior. Health students had higher knowledge about health because they frequently participated in clinical and public health practice and training^(16)^. The nursing students have a high understanding of COVID-19 because of their educational background, information from social media, and attending health seminars or training^(17)^. Knowledge is reflected in appropriate attitudes and practices because knowledge can affect professional readiness and creates positive behavior^(18)^. However, even after adequate knowledge, the attitude was not always positive, requiring additional education to emphasize the necessity of developing a positive attitude and continuous preventive action to decrease COVID-19 transmission^(19)^. Educational interventions were effective in preventing respiratory infections in adults^(20)^.

Attitude is defined as the tendency to act, which means that attitudes were part of behavior, how individuals behave can reflect how they feel^(21)^. Knowledge and attitude have a positive linear relationship^(22)^. Positive knowledge influences a person’s positive attitude toward COVID-19 transmission prevention behavior, so someone with a positive attitude is influenced by positive knowledge^(22)^. Sanyod *et al*. discovered that nursing students had a positive attitude toward COVID-19 because they had more knowledge and information about COVID-19 and had performed direct care in hospitals to treat positive COVID-19 patients^(23)^. So, nursing students are well prepared to prevent COVID-19. The study carried out by Saglain *et al*. found that attitudes did not differ significantly with age, gender, experience, or profession^(22)^.

The primary risk factor for disease spread is behavioral influence^(24)^. In the face of critical health situations, compliance with preventive behaviors is strongly influenced by individual factors, cultural characteristics, beliefs, norms, socioeconomic, political, territorial, and the perception of risk associated with illness^(24)^. Personal positive behavior cannot be separated from the government’s role, community, and health workers, who are continuously attempting to educate people through the media and develop community policies and guidelines for preventing COVID-19 transmission. When one person receives societal support, he or she is expected to behave in a certain manner^(25)^. People’s expected behaviors may be supported by adequate facilities. On the questionnaire, all respondents agreed that the availability of vaccines provided by the faculty/university was the primary reason for their participation in vaccination. The presence of face masks, as well as facilities to wash hands with soap and running water in several public places, such as dorms and practice sites, demonstrates the availability of facilities. Adequate behavior to prevent COVID-19 transmission can aid in COVID-19 suppression. As a result, practicing daily hand hygiene, wearing masks, and keeping a social distance will have a positive impact on the current pandemic.

The findings revealed that there was no correlation between the level of knowledge and the behavior in preventing COVID-19 transmission. This can be caused by respondents being third-year nursing students, the majority of whom are females aged 20 and up, all of which influence Covid-19 prevention behavior. Factors such as demographic conditions such as gender, age, length of study, place of residence, and institution type all had a significant impact on the practice of preventing COVID-19 transmission^(12-13,17,26)^. The other studies revealed that students in the age group over 20 have higher knowledge, attitude, and practice scores toward COVID-19 than students under 20 years of age, and that these differences are statistically significant for both male and female students^(12,17)^.

Female students and students over the age of 20 were found to have high knowledge, a positive attitude, and good behavior^(12,17)^. Gender is an important variable influencing prevention behavior, so women show better adherence than men probably because they have greater motivation for health than men^(27)^. This could be due to female students having a higher internal locus of control and learning motivation than male students, allowing them to achieve higher knowledge and practice scores^(12)^. Sondakh *et al*. discovered that students over the age of 20 have higher knowledge, attitudes, and practices than those under the age of 20^(12)^. Older nursing students (age ≥ 20 years old) typically gain more knowledge and experience as a result of longer exposure to learning (curriculum) and practical experience^(12)^. Students’ knowledge improved as they progressed up the academic ladder, possibly because they were exposed to high-level studying and COVID-19-related information in their courses and clinic exposures^(17)^. As a result, it is not surprising that older nursing students have better knowledge, attitudes, and practices regarding Covid-19 prevention than younger nursing students (age <20 years old).

In addition, knowledge will be able to influence behavior by building self-efficacy in advance of the related behavior. Another study found that self-efficacy action is associated to wear a face mask and wash one’s hands, and that action control helps to bridge the gap between intention and behavior^(28)^. So, even if someone has a high level of knowledge, it was not always put into practice because other factors are required, such as the ability to meet needs, the availability of facilities, motivation, and support from close friends and family. An important concept to understand is that past habits have a significant influence on a person’s changes in behavior. Past habits that completely or partially do such behaviors are discovered to possibly inhibit the attainment of new expected behaviors^(29)^.

The study’s findings revealed a significant correlation between attitudes and behavior in COVID-19 transmission prevention, even with a high correlation strength. Based on this study, these results can be explained by most participants’ high level of knowledge, female and aged over 20. In addition, the government regulation enforced restrictions on communal activities to prevent the spread of the pandemic. High levels of knowledge have been shown to influence positive attitudes and behaviors^(22)^. According to an others study, improved infection behavior prevention can be more adhered to when one has a good attitude toward COVID-19 prevention^(13,30)^. Respondents with a positive attitude will exhibit good prevention behavior^(31)^. The more positive the student’s attitude, the more positive the student’s behavior^(11)^. Rachmani *et al*. discovered that respondents who did not follow health protocols correctly were more likely to be negative in their attitudes^(32)^. Thus, it can be concluded that nursing students’ positive behavior in preventing COVID-19 is influenced by their positive attitude toward it.

### Study limitations

The authors recognize that there are limitations, such as the use of the accidental sampling method in this study, which means that the sample obtained does not represent the population and is prone to selection bias or errors in the selection process if further selection was not performed. A further limitation of online survey research is self-selection bias.

### Contributions to Nursing, Health, or Public Policy

The study’s findings can be used by educational institutions and healthcare settings to assist nursing students in developing and maintaining positive attitudes and behaviors toward COVID-19 prevention through continuous education programs, policies, health resources, and the provision of supporting facilities for COVID-19 prevention following health protocols.

## CONCLUSIONS

Most third-year nursing students at a private university have a high level of knowledge, a positive attitude, and positive behavior toward preventing COVID-19 transmission. This is related to the amount of health information nursing students receive from lectures, literature, health seminars, training, and practical experience. In addition, knowledge level was found to be unrelated to COVID-19 prevention behavior. This could be because other factors such as demographics, facilities, motivation, and support have an impact. Furthermore, attitudes were associated with COVID-19 prevention behavior in nursing students because attitudes became a tendency to act. The better the student’s attitude, the better the student’s behavior.

Nursing students are expected to maintain positive attitudes and behaviors toward COVID-19 prevention as future health workers at the forefront of health services. Additional research can investigate other factors that influence COVID-19 prevention behavior, such as motivation, availability of facilities, support, and policies.
